# Combining Stochastic Resonance Vibration With Exergaming for Motor-Cognitive Training in Long-Term Care; A Sham-Control Randomized Controlled Pilot Trial

**DOI:** 10.3389/fmed.2020.507155

**Published:** 2020-11-30

**Authors:** Eling D. de Bruin, Heiner Baur, Yvonne Brülhart, Eefje Luijckx, Timo Hinrichs, Slavko Rogan

**Affiliations:** ^1^Department of Health Sciences and Technology, Institute of Human Movement Sciences and Sport, ETH Zurich, Zurich, Switzerland; ^2^Division of Physiotherapy, Department of Neurobiology, Care Sciences and Society, Karolinska Institute, Stockholm, Sweden; ^3^Department of Health Professions, Bern University of Applied Sciences, Bern, Switzerland; ^4^Division of Sports and Exercise Medicine, Department of Sport, Exercise and Health, University of Basel, Basel, Switzerland

**Keywords:** aging, physical performance, executive functions, whole-body vibration, exergame, motor-cognitive training, long term care, frail adults

## Abstract

**Purpose:** Physical and mental functions allow classifying older adults as “Go-Go” (independent functioning); “Slow-Go” (in need of care with a slight handicap); and “No-Go” (in need of care with severe functional limitation). The latter group exhibits reduced exercise tolerance. More recently technology-based motor-cognitive types of training services emerged as a possible training service. This study examined the use of technology including stochastic resonance whole-body vibration and Exergame-dance training for motor-cognitive training in care home dwelling adults.

**Methods:** Seventeen older adults (10 women, 7 men, age range: 79–98) were randomly assigned to the intervention (IG, *n* = 9) or the sham group (SG, *n* = 8). IG performed five sets of 1-min whole-body vibration with 1-min rest in between, three times a week for the first 4 weeks of the training period with varying frequency. From weeks five to eight the Exergame-dance training was conducted after the vibration sessions. SG performed a stochastic resonance whole-body vibration training with the same terms applied, however, with a fixed frequency of 1 Hz, Noise 1. From weeks five to eight a passive trampoline-programme of 5 min was applied following the vibration sessions. Primary outcome was the Short Physical Performance Battery (SPPB). Secondary outcomes were the Trail Making Test A and B (TMT A & B) and the Falls Efficacy Scale–International (FES-I). Outcomes were measured at baseline, after 4 and 8 weeks of intervention and at follow-up (4 weeks after the intervention). The non-parametric Puri and Sen rank-order test was applied, followed by an ANOVA for repeated measures to analyse main and interaction effects. Mann–Whitney *U*-Test was used to determine differences between the groups.

**Results:** The *post-hoc* analysis showed significant effects on the SPPB total score with large effect sizes from baseline to 8 weeks (+72%, *p* = 0.005, η^2^ = 0.423). The TMT part B displayed significant improvements with large effect sizes from baseline to 8 weeks (+17.5%, *p* = 0.002, η^2^ = 0.779) and to follow-up (+21%, *p* = 0.001, η^2^ = 0.827).

**Conclusion:** The technology based 8-week training programme consisting of a combination of stochastic resonance whole-body vibration and Exergame-dance training showed beneficial effects on both physical and cognitive performance in older care home dwelling adults.

## Introduction

Maintenance or expansion of intellectual powers, autonomy, and superior physiological functioning, characterizes successful aging (SA) (1). SA leads to enhanced health and ability, and associates with avoiding or even reversing of losses in functioning. Improvement of physical fitness, consuming good nutrition, and maintaining normal weight all are moderating factors that can be used for prevention and help in turning usual into successful aging processes (1).

Usual aging is associated with reduced autonomy, poor physiological functioning, the loss of balance, and cognition (1, 2) as well as a general decline in functional performance (3). Decline in functional performance includes loss of muscle strength, muscle mass, and physical capacity. An accumulation of physical limitations, cognitive impairments, and/or loss of social support may drive functional decline to such an extent, that institutionalization in long-term care becomes a reality (4). Due to “The Paradox of Nursing Home Funding” (4), resources for long-term care facilities are greater when the functional status of the residents is worse, which may explain the lack of rigor regarding available rehabilitation options offered to residents of long-term care facilities (4). As a consequence sarcopenia-related mortality in long-term care is rather prevalent (5) which calls for improved prevention efforts in long-term care settings. Exercise programmes are needed that enable maintenance of functional performance; e.g., through strengthening of the leg extensor muscles (6), at the highest possible level in long-term care to enhance independence and lower the risk of developing or aggravating disability (7).

Narrative reviews (8, 9), and an experts report giving guidance to workers in long-term care (10), suggest the use of novel technologies to be considered for the enhancement of physical activity in long-term care settings. Older long-term care dwellers that are incapable to conduct conventional types of training and exercise, because of their limited remaining strength and physical capacity (11), might be motivated and supported with the help of technology-supported exercise solutions. However, currently there is a lack of exercise studies in long-term care in general (12) and, due to the novelty of many technological approaches, in particular for technology-supported exercise programs.

Based on their physical and mental functions older adults may be classified into three categories: 1. “Go-Go” = independent functioning older individuals; 2. “Slow-Go” = Older adults in need of care with a slight handicap; and 3. “No-Go” = older individuals in need of care with severe functional limitation; e.g., frail and mobility disabled older adults (13). “No-Go” or frail older persons have difficulties with their neuromuscular functions, often impeding them from participating in conventional strength and endurance trainings (14). No-Go older adults that are not able to perform traditional forms of exercise because of too low physical capacities are, however, amenable to whole body vibration (WBV) (15). It can, therefore, be hypothesized that WBV is applicable as a “skilling-up” exercise for older adults with low physical capacity (i.e., the No-Go group), where a main focus is put on the activation of the neuromuscular system (16). WBV effectively and reliably enhances mobility, strength and balance and helps avoiding falls (17, 18). The only requirement of WBV to be applicable is to be able and stand in vertical position on a vibrating platform for 1 min. Benefit of WBV is its low impact on the body, while firing multiple impulses to the neuromuscular system (14, 19, 20).

Recent literature supports the notion that alongside with the decrease of sensorimotor functions, cognitive decline associates with the risk of falling (21–26) and gait control (27). Hence, cognitive elements should be part of a training programme for elderly individuals since these can enhance physical functions such as walking (28, 29). Since virtual games and virtual realities (VR) effect both on sensorimotor and cognitive functions (28, 30–32), combining exercise with videogaming is a viable option to achieve the goal of effecting on cognition. Pilot studies from our group with SR-WBV and Exergame-dance training (EXDT) as motor-cognitive training showed promising results in frail older long-term care home dwellers as being a safe and well-accepted intervention effecting on balance (11, 33). Furthermore, training with individually adaptable Exergames is applicable in people with mild cognitive impairment (34) or with major neurocognitive disorders (35, 36).

In view of the above, the aims of this study were to assess the effects of SR-WBV that gets combined with ExDT on (i) functional and (ii) cognitive performance of care-dependent older adults. An 8-week intervention consisting of a combination of SR-WBV and ExDT was developed and assessed. We hypothesized that the combination of SR-WBV and ExDT would have beneficial effects on functional and cognitive performance of care-dependent older trainees.

## Materials and Methods

### Study Design

This study was an experimental double-blind randomized controlled trial with participants randomly assigned to either an intervention or a sham group (SG). A sham control treatment is used for non-pharmacological studies looking at devices and psychological and physical treatments (37). In our trial the trainees went through the same motions on the vibration plate. During the active phase of the training similar movements were expected on the dance plate and the trampoline. In both instances, however, the sham group did not receive the active ingredient of the training; vibration intensity level or the active interaction with the screen monitor required to perform the game.

**Figure 1:**
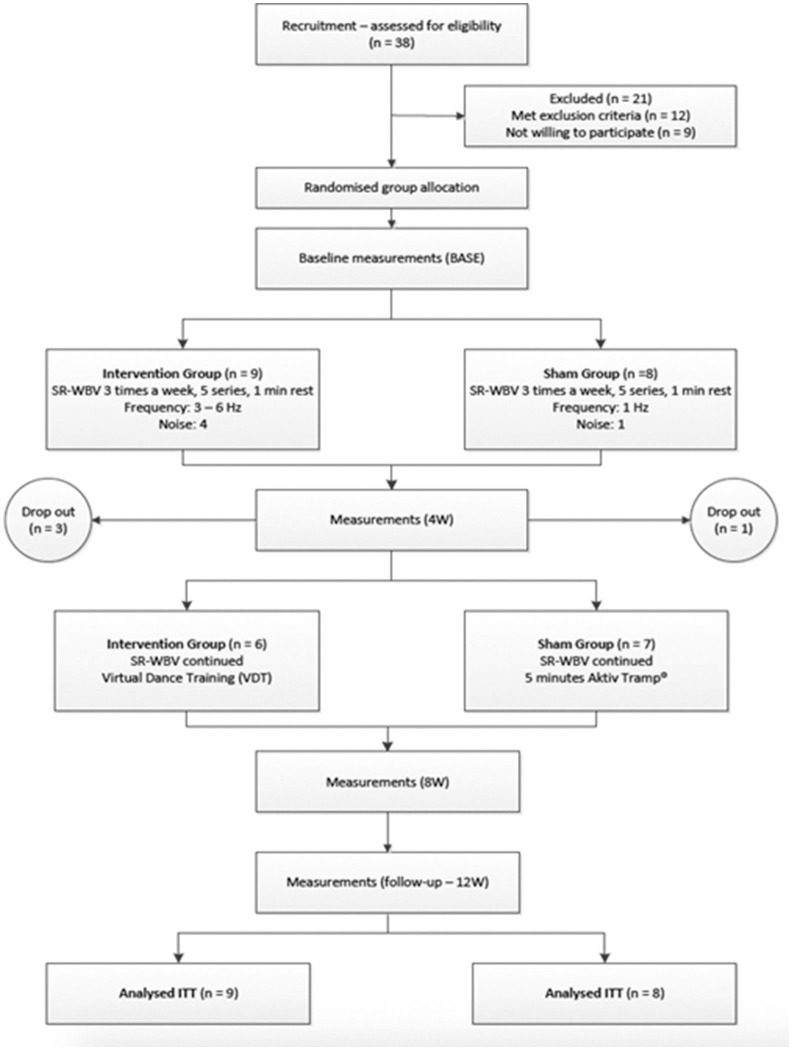
Study flow chart of the allocation and the participants throughout the study. SR-WBV, stochastic resonance whole-body vibration; 4W, after 4 weeks; 8W: after 8 weeks; 12W: after 12 weeks.

Assessor and study participants were blinded. The assessor carried out the measurements without information about participant group allocation. The participants were informed about two possible training interventions with different intensities during the information session, however, were not informed about the details underlying the intensity differences. Measurements were carried out at baseline (BASE), after 4 weeks (4W), and after 8 weeks (8W). Follow-up data were collected 4 weeks after the intervention end (follow-up; Figure 1). CONSORT guidelines for randomized trials were followed for reporting (38).

### Participants

Recruitment took place in a nursing home in Horgen, Zurich, Switzerland. The target population consisted of 17 care-dependent residents of the nursing home, aged 65 years or older. Care dependence was defined by whether the older adults permanently depended on assistance or support in their everyday activities (e.g., body care, dressing, eating, use of toilet, mobility, planning the day) (39). Older adults in need of care were included if they were able to stand for 1 min with or without aids, reached a minimum of 16 points in the Mini Mental State Examination Test (MMSE) and had a point score of 6 or below in the Short Physical Performance Battery (SPPB). In addition, the doctor of the nursing home decided whether the participants were resilient enough to partake in the intervention. Resilience was defined as “a dynamic process of maintaining positive adaptation and effective coping strategies in the face of adversity”(40) and judged on clinical experience from the nursing home MD. Exclusion criteria were: severe visual problems, acute fractures or thrombosis, epilepsy, migraine headaches, acute back pain, or active arthritis and amputation of the lower limb.

### Randomization Procedure

An independent statistician performed the randomization, using an Excel table (Microsoft® Excel® for Mac, 2011). After the baseline measurements (BASE) the participants were randomly assigned to either the IG or the SG by means of sealed opaque envelopes that were distributed prior to the start of the intervention phase. Envelopes were opened sequentially and only after participants' details were written on the envelope.

All participants received oral and written information prior to the recruitment and written informed consent was obtained. The study protocol has been approved by the Ethics Committees from Cantons Bern and Zurich (EK ZH No 2014-0469) and was registered at NCT02332083 (https://clinicaltrials.gov/ct2/show/NCT02332083?term=NCT02332083&draw=2&rank=1).

### Intervention Protocol

Each participant was familiarized with the SR-WBV equipment prior to the start of the intervention. Based on the group allocation participants were familiarized with the Exergame equipment or the Aktiv Tramp® that was used for the control training group.

### Stochastic Resonance Whole-Body Vibration (SR-WBV)

**Figure 2:**
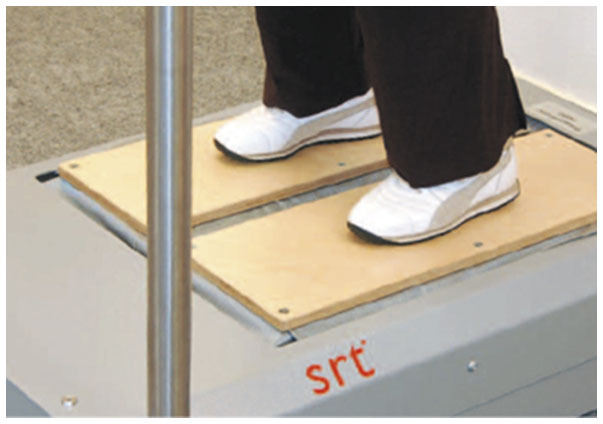
Zeptor med® device. Example of the vibration training on the Zeptor med® device with two feet on separate platforms.

All participants were trained to take the standardized standing position on the Zeptor med® plus Noise (Frey AG, Zurich, Switzerland; Figure 2): wearing no shoes, stand with parallel feet and with slightly bent ankle, knee, and hip joints. WBV with stochastic resonance (SR-WBV) required from the individuals to stand on two separate randomly oscillating platforms. The platforms independently move up/down, forward/backward, and right/left with an amplitude of 3 mm. Stochastic resonance can be defined as a “non-linear cooperative effect” where the addition of a random process or noise to a weak signal results in the phenomenon of stochastic resonance (41). Low-level mechanical or electrical noise transferred to sensory neurons can significantly enhance their ability to detect weak signals (42). Weak signal frequencies between 1 and 12 Hz can be adjusted on the device. The selectable noise levels range from 1 (weak interference function) to 5 (strong). Participants could slightly hold on to the rails on one or both sides for security.

The intervention was carried out on 3 days per week over a period of 8 weeks leading to a total of 24 sessions. The training in the first 4 weeks consisted of five 1-minute-vibration sessions followed by a break of 1 min. The IG started with a basic frequency of 3Hz and a noise level 4. The exercise was intensified in small increments: if the participants were able to stand parallel without holding on to the rails, the frequency was enlarged by 1Hz. If 6Hz were accomplished, the difficulty of the standardized standing position was increased (tandem standing position, slow dynamic functional squat movements). Each foot stood on one platform and the same augmentation criteria as written above were applied. This progression was applied according to the procedure of Kessler et al. (3). SG vibrated with a frequency of 1Hz and a noise level of 1 without progressing over the course of training regarding adapting the body standing position. The frequency and constant position have previously shown no effects (11, 43) and, thus, were deemed suitable as sham intervention.

### Exergame-Dance Training (ExDT)

**Figure 3:**
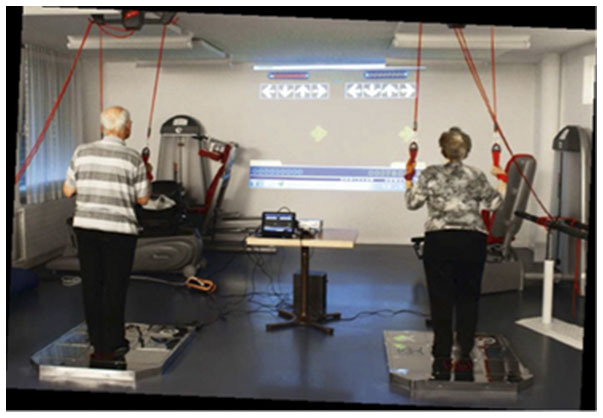
Step Game. Example for the virtual dance training with the Step Game beamed on the wall.

**Figure 4:**
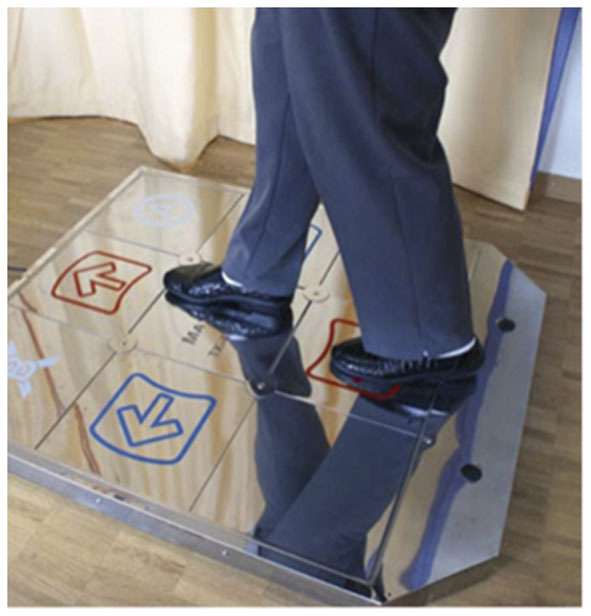
Dance pad. Example of the virtual dance training on the dance pad.

ExDT was performed by the IG on a step plate (Dance Pad DDR, 93 × 14.7 × 109 cm, Mayflash) and combined with Step Game Software (StepMania 4.0). The Step Game scenario, that has shown to be feasible and safe when applied in vulnerable populations (36, 44), was projected on a wall using a commercially available video beamer (Figure 3). Arrows moved from different directions toward target squares pointing up, down, left, and right. Trainees were expected to step on the arrow positions of the platforms, when arrows reached the target squares in time with the music (32 to 137 beats per minute) (Figure 4). As the performance levels increased, additional distracting shapes that crossed the screen were added. In these cases; e.g., when triangles instead of arrows appeared, the participants were expected not to react by stepping on the target zones. Electronic sensors in the step plate detected position and timing information that was then used to provide participants with real-time visual feedback on performance. The participants completed a training session of ~5 min. Development of performance was controlled through the points achieved per difficulty level. Details about the training were previously reported (45). Scores relate to steps that had to be performed exactly when an arrow reached a highlighted area on the screen to achieve best scores in the game. In case of hitting a note outside of the best score window, but within the timeframe of that next lower level you gain a lower point judgement. Levels of difficulty in stepping patterns and frequency were created and different styles of music were chosen to add variety and meet participants' preferences. This approach meets the specifics for training postural control (46) and the postural modality of the exercises in combination with the required spatial processing demands (47) enhances both processing speed and attentional selectivity (48).

### Aktiv Tramp®

**Figure 5:**
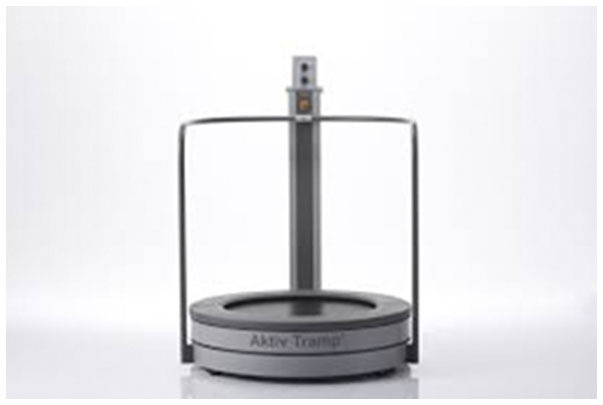
Picture of the Aktiv Tramp® (Frei Swiss AG).

The Aktiv Tramp is a trampoline with frequency control. Adjustable vibrations are transmitted to the trampoline by means of a motor. The trampoline swings up and down and the trainee is expected to follow these swings. SG performed their sham training on the Aktiv Tramp® (Frei Swiss AG, Thalwil, Switzerland) (Figure 5). The frequency with which the trampoline vibrates can be adjusted, similar to the Zeptor med®. Participants stood on the trampoline without shoes for 5 min. Both hands were positioned on the rail. The frequency was set at 1 Hz. To the best of our knowledge no study analyzed and reported the effects of the Aktiv Tramp® on physical function or cognitive performance. Since a frequency of 1Hz on the Zeptor med® showed no effects (11, 43), we assumed this type of training to not have effects.

### Primary Outcome Short Physical Performance Battery

The Short Physical Performance Battery (SPPB) (49) is a clinical test battery measuring lower extremities physical performance. It is highly reliable (50) and consists of three single test components: “balance” (side-by-side, semi- tandem and tandem stance), “gait speed” and “five times chair rises.” Twelve points are the maximum possible test score. A SPPB total score from 0 to 6 points is a “weak,” 7–10 points a “mean” and 11–12 is a “good” performance (51). The threshold for fulfilling the criteria of “being frail” in elderly in need of care was set on a SPPB total score of ≤6 points (52), a score associated with falls (53), functional disability (54), and an increased risk of all-cause mortality (55). Furthermore, the SPPB is predictive for the onset of difficulty in basic activities of daily living and most highly recommended for the identification of older adults at risk for the loss of independence (56, 57). Each test was scored individually as a single item score. Each single test was scored from zero (worst performance) to four (best performance).

### Secondary Outcome Trail Making Test A and B (TMT-A and -B) and Falls Efficacy Scale-International (FES-I)

Cognitive performance was assessed with the Trail Making Tests A & B (TMT-A, TMT-B) which is often used to assess exercise-cognition interaction (58). The TMT is a neuropsychological test that verifies visual attention and executive function (59). It also provides information about psychomotor processing speed (60) and cognitive flexibility (61, 62). TMT-A depends on visual-perceptual abilities and has little executive input, TMT-B reflects working memory and task-switching ability and stresses central executive processes, and TMT B-A is an indicator of central executive function (58, 63). Results of Trail A and Trail B were reported as the number of seconds required to complete the test. A higher score (Trail A: >78 s; Trail B: >273 s) is associated with a higher risk of injurious falls and poorer physical performance (60).

To assess the concern about falling, the Falls Efficacy Scale-International (FES-I) was deployed. With a Cronbach‘s α = 0.96 the FES-I showed excellent internal consistency and has, with an ICC = 0.96, high test-retest-reliability (64). The German language FES-I was used in this study (65). The FES-I has a minimum of 16 points (no concern about falling) and a maximum of 64 points (high risk of falls).

### Statistical Analysis

Adjusting to the small sample size, non-parametric analysis procedures were applied. IBM SPSS Statistics 22 for Windows (SPSS, Inc., Chicago, Illinois, United States) and Microsoft® Excel® for Mac 2011 were used for the statistical analysis.

All available data were analyzed with an intention-to-treat-approach (66). Each participant that started the intervention was included in the analysis, regardless of the adherence rate (67). We assumed that all missing data were constant and replaced the missing values with the mean values of their allocated group (68).

The non-parametric rank-order tests of Puri and Sen L Statistics were used for statistical analysis (69). This method includes two steps; (i) the data were converted into ranks; (ii) a two-way analysis of variance (ANOVA) with repeated measures was applied, examining the between-group main effects (BASE, 4W, 8W) and the group-by-time interaction (IG, SG).

Pillai's trace was used to calculate L. α-level was set at *p* < 0.05. In case of statistical significance, Mann–Whitney-*U*-tests (α-level: *p* < 0.0125, after Bonferroni correction) were used for *post-hoc* analysis. In addition, the effect sizes (ES) for the post-training and follow-up effects within and between groups, eta-squared (η^2^) were calculated. For η^2^, an effect size of 0.01 is considered a “small” effect, around 0.06 a “moderate” effect and 0.14 and above a “large” effect (70).

## Results

### Baseline Comparisons and Participant Flow

No difference between the IG and the SG was found in any of the subject demographic and anthropometric characteristics (Table 1). Thirty-eight participants were assessed for eligibility. Twelve out of these 38 did not meet the inclusion criteria and nine were not willing to participate. Seventeen participants met all the inclusion criteria and were willing to participate. Figure 1 shows the participant flow through the study. A total number of 13 participants completed the intervention, resulting in an adherence rate of 76.5%. These 13 participants completed 100% of the 24 scheduled training sessions. All data presented resulted from the original intention-to-treat analysis. There were no adverse events.

**Table 1:** Demographic and anthropometric characteristics of the participants.

	**IG (*n* = 9)**	**SG (*n* = 8)**	***p***
Sex (female/male)	5/4	5/3	
Age (years, mean, ± SD)	86.1 ± 5.9	90.3 ± 5.9	0.29
Age (range)	79–98	80–95	
Height (m, mean, ± SD)	1.66 ± 0.04	1.62 ± 0.06	0.10
Weight (kg, mean, ± SD)	68.4 ± 15.2	65.1 ± 10.3	0.34
MMSE	26.6 (21.75–30.00)	28.0 (25.00–29.00)	0.86
SPPB	3.5 (2.75–5.25)	4.0 (2.00–5.00)	0.83

*IG, intervention group; SG, sham group; SD, standard deviation; MMSE, Mini Mental State Examination Test; SPPB, Short Physical Performance Battery*.

### Primary Outcome SPPB

The SPPB baseline measurements total score and single test item scores showed no differences between IG and SG (**Table 3**). The repeated measures ANOVA of the SPPB total score and single items score over the intervention and follow-up time after 12 weeks of SR-WBV, ExDT, and Aktiv Tramp® showed significant differences for the SPPB total score between-group main effects. Significant between-group main effects for the SPPB single item chair rises and the SPPB item balance were displayed (Table 2).

**Table 2:** SPPB ANOVA with repeated measurements (ranks) between-group main effects and group-by-time interaction.

	**Pillai‘s trace (*r^**2**^* = SS_**Bet**_/SS_**Tot**_)**	**L [(N-1) *r^2^*]**	**p**	**ES (η^2^)**
SPPB total score (main effects)	1.80	7.79	0.003^*^	0.64
SPPB total score (interaction effects)	0.76	3.28	0.055	0.43
SPPB chair rises (main effects)	0.65	7.86	0.003^*^	0.65
SPPB chair rises (interaction effects)	0.51	4.58	0.021^*^	0.51
SPPB gait speed (main effects)	0.27	1.62	0.232	0.27
SPPB gait speed (interaction effects)	0.22	1.25	0.332	0.22
SPPB balance (main effects)	1.45	6.30	0.007^*^	0.59
SPPB balance (interaction effects)	0.35	1.55	0.248	0.26

SPPB, Short Physical Performance Battery;

* *significant difference p < 0.05, ES, effect size (η^2^ = 0.01; small effect, η^2^ = 0.06; moderate effect, η^2^ = 0.14; large effect)*.

The result of the *post-hoc* analysis is presented in Table 3. Participants of the IG improved their SPPB total score significantly from BASE to 8W by 2.8 points, participants of the SG by 0.5 points. The chair rises single item score improved from 0.9 to 1.7 points from BASE to follow-up in the IG, by 0.2 points in the SG. The SPPB balance single item score from BASE to 8W was improved by 1 point in the IG and did not change in the SG. The SPPB gait speed single item score improved by 1 point in IG from BASE to follow-up, whereas SG showed a change of 0.3 points in this item.

**Table 3:** SPPB *post-hoc* analysis at BASE, 4W, 8W, and follow up.

	**BASE**	***p***	**4W**	***p***	**8W**	**p ES (η^2^)**	**Follow up**	**p ES (η^2^)**
SPPB total score IG (*n* = 9)	3.9 ± 1.5	0.729	5.0 ± 1.5	0.143	6.7 ± 1.6	0.005^*^	6.9 ± 1.6	0.018
SPPB total score SG (*n* = 8)	3.6 ± 1.5		3.9 ± 1.6		4.1 ± 1.6	0.423	4.4 ± 1.8	0.368
SPPB chair rises IG (*n* = 9)	0.9 ± 1.1	0.915	0.9 ± 1.1	0.540	1.7 ± 1.3	0.236	1.9 ± 0.9	0.165
SPPB chair rises SG (*n* = 8)	0.8 ± 0.5		1.1 ± 1.0		1.0 ± 0.8	0.109	1.1 ± 1.0	0.141
SPPB gait speed IG (*n* = 9)	1.3 ± 0.7	0.765	1.6 ± 0.7	0.507	2.3 ± 0.8	0.080	2.0 ± 0.7	0.033
SPPB gait speed SG (*n* = 8)	1.3 ± 0.9		1.3 ± 0.7		1.6 ± 0.9	0.162	1.1 ± 0.8	0.262
SPPB balance IG (*n* = 9)	1.7 ± 0.9	0.870	2.5 ± 0.7	0.004^*^	2.7 ± 0.8	0.021	2.9 ± 0.3	0.168
SPPB balance SG (*n* = 8)	1.6 ± 0.9		1.4 ± 0.5		1.6 ± 0.9	0.329	2.1 ± 1.1	0.188

IG, intervention group; SG, sham group; SPPB, Short Physical Performance Battery; BASE, baseline measurements; 4W, after 4 weeks; 8W, after 8 weeks;

* *significant difference after Bonferroni adjustment p < 0.0125; ES: effect size (η^2^ = 0.01; small effect, η^2^ = 0.06; moderate effect, η^2^ = 0.14; large effect). Results are expressed as SPPB point scale in mean and standard deviation (±)*.

### Secondary Outcomes TMT-A and -B and FES-I

The baseline measurements of the TMT A, B & B minus A showed no significant differences between IG and SG (**Table 5**). The repeated measures ANOVA of the TMT part B over the intervention and follow-up time after 12 weeks of SR-WBV, EXDT, and Aktiv Tramp® showed significant differences for the group-by-time interaction with a large ES. TMT B minus A showed a main effect of time and a significant interaction between the two groups (Table 4). The results of the *post-hoc* analyses of the secondary measurements are presented in Table 5. The TMT part A showed no significant differences in the ANOVA and can therefore be neglected for further analysis. Participants of the IG improved their TMT part B by 18% from BASE to 8W and by 21% to follow-up, whereas the participants of the SG showed a negative change of −16 and −18% in this outcome. TMT B minus A revealed a group difference in week 4 only.

**Table 4:** ANOVA with repeated measurements (ranks) between-group main effects and group-by-time interaction for the secondary outcomes TMT A & B and FES-I.

	**Pillai's trace** **(*r^**2**^* = SS_**Bet**_/SS_**Tot**_)**	**L [(N-1) *r^**2**^*]**	***p***	**ES (η^2^)**
TMT A (main effects)	0.43	1.85	0.189	0.299
TMT A (interaction effects)	0.07	0.32	0.814	0.068
TMT B (main effects)	0.01	0.02	0.995	0.005
TMT B (interaction effects)	1.58	6.84	0.005^*^	0.612
FES-I (main effects)	0.001	0.004	1.000	0.001
FES-I (interaction effects)	0.287	1.244	0.334	0.22

TMT, Trail Making Test; FES-I, Falls Efficacy Scale-International;

* *significant difference p < 0.05; ES, effect size (η^2^ = 0.01; small effect, η^2^ = 0.06; moderate effect, η^2^ = 0.14; large effect)*.

**Table 5:** *Post-hoc* analysis at BASE, 4W, 8W, and follow up for the secondary outcomes TMT A & B and FES-I.

	**BASE**	***p***	**4W**	***p***	**8W**	***p* ES (η^2^)**	**Follow up**	***p* ES (η^2^)**
TMT A IG (s), (*n* = 9)	71.9 ± 38.6	0.336	69.8 ± 32.43	0.054	73.8 ± 44.6	0.248	68.7 ± 44.9	0.700
TMT A SG (s), (*n* = 8)	111.7 ± 79.0		124.5 ± 75.63		116.0 ± 83.8	0.157	66.5 ± 17.8	0.116
TMT B IG (s), (*n* = 9)	199.3 ± 55.0	0.594	206.0 ± 53.3	0.239	164.3 ± 44.5	0.002^*^	157.5± 27.6	0.001^*^
TMT B SG (s), (*n* = 8)	197.7 ± 33.1		209.7 ± 48.5		231.1 ± 46.5	0.779	233.1 ± 0.0	0.827
TMT B-A IG	132.6 ±31.2	0.174	151.7 ± 31.2	0.040^*^	124.4 ± 38.4	0.621	117.7 ± 72.0	0.45
TMT B-A SG	67.3 ± 87.9		93.0 ± 54.7		148.5 ± 86.8	0.023	150.3 ± 82.1	0.049
FES-I IG	34.2 ± 10.3	0.961	22.8 ± 4.4	0.627	22.2 ± 4.9	0.439	20.7 ± 3.2	0.244
FES-I SG	35.6 ± 14.9		28.4 ± 16.6		31.1 ± 18.2	0.169	34.4 ± 19.1	0.190

Results are expressed as mean and standard deviation (±). IG, intervention group; SG, sham group; TMT A & B, Trail Making Test A & B; FES-I, Falls Efficacy Scale—International; BASE, baseline measurements; 4W, after 4 weeks; 8W, after 8 weeks; s, seconds;

* *significant difference after Bonferroni adjustment p < 0.0125; ES, effect size (η^2^ = 0.01; small effect, η^2^ = 0.06; moderate effect, η^2^ = 0.14; large effect)*.

The baseline measurements of the FES-I showed no significant differences between the IG and the SG (Table 5). The repeated measures ANOVA of the FES-I over the intervention and follow-up time after 12 weeks of SR-WBV, EXDT, and Aktiv Tramp® showed no significant differences and can therefore be ignored for further analysis (Table 4).

## Discussion

This experimental double-blinded randomized controlled trial examined the effects of an 8-week intervention with SR-WBV combined with EXDT on physical functional performance and cognition in care-dependent elderly. Our results show that the combination of SR-WBV and EXDT has beneficial effects on both physical and cognitive performance levels when compared to a sham intervention.

The SPPB physical performance measure used in our study is an objective tool evaluating lower extremity function. Poor performance on the SPPB is a powerful predictor of adverse health outcomes, such as increased need for support from caregivers, functional decline, institutionalization, and mortality (49, 51). The results of the present study showed significant beneficial effects on lower extremities functional performance of the technology-based training group. The SPPB total score and the single items gait and balance improved significantly in the IG after the intervention and these positive effects were still persistent at follow-up. The IG improved by 2.8 points on the SPPB total score from baseline measurement to 8 weeks, the sham training group changed by 0.8 points only. An improvement of one point on the 12-points SPPB scale is labeled as “considerable” (71) or “substantial meaningful” (72) change. A low SPPB score is, furthermore, predictive for lower activities of daily life and increased disability (51), decreased independence (50), loss of mobility (73), and increased mortality (74, 75). Therefore, an improvement of the SPPB total score as seen in the training group may reduce the risk of disability and mortality of long-term care dwellers and enable them to live more actively and independently.

Loss of muscle mass in older age is associated with the loss of strength and power (76–78). Gait speed and repeated chair rise capacity are associated with the loss of strength and independent functioning (6). Studies suggest that gait speed is the most sensitive subtask of the SPPB in predicting incident disability (79, 80) and chair rise performance in predicting mortality (75, 81). Our study displayed that a technology-driven intervention consisting of SR-WBV and EXDT improves both walking and chair rising in the long-term care dwelling “No-Go” -trainees. Our data are in line with other research showing the positive effect of physical rehabilitation in long-term care dwellers, however, adds novelty in the sense that we reach large effects of training in rather weak and frail residents that are mostly excluded from exercise interventions (82).

The findings concerning improvements of physical function support previous studies examining the effect of WBV training in the elderly (3, 14, 17–43, 43–86). Pollock et al. (14) found significant improvements in mobility following 8 weeks of WBV training with elderly. The effects might be due to the sequential offering of the WBV and EXDT components. Stochastic stimuli at already low intensities are able to affect the membrane potential of nerve cells which leads to an activation of the neuromuscular systems (87) and prepares the trainees for the second training component. The EXDT adds the training of locomotor control to the WBV component. Trainees are expected to generate motor commands to activate their muscles and, by doing that, act on the external environment (pressure sensitive platform). They visually sense their reaction shown on the virtual (flat screen) environment and this information is fed back to their central nervous system and allows adjustments of motor commands. This training approach reflects an integrative approach of how locomotor systems function (88), and aims to improve an individual's functions by tapping on the domains of Intrinsic Capacity (IC) (89), “the composite of all the physical and mental capacities of a person (90).”

Aerobic (91–93) and strength training exercise (94, 95) benefit cognition; e.g., especially executive functions. Our study shows that a combination of SR-WBV and EXDT also has significant beneficial effects on cognitive performance in rather weak care-dependent elderly. The TMT part B, that requires more executive skills (62), showed significant enhancement with concomitant large effect size following the 8 weeks intervention. These results support the hypothesis that a training programme for elderly should consider both functional and cognitive elements (9, 11, 28, 31, 68, 96) and is in line with other research showing improved executive functioning following exergaming (32, 97, 98). Because impaired executive functions relate to a higher risk of (repeated) falling, training programs should also target this cognitive domain (61, 99, 100). When the TMT B minus A values are observed the changes are not consistent. Following 4 weeks of training a significant change is observed, however, this is not maintained in the subsequent assessments. TMT B-A is an indicator of central executive function (58, 63) and, thus, these results may indicate that training intensity and/or duration was not sufficient to effect on central executive function. However, TMT A and B are substantially correlated measures which causes low levels of reliability for the differences (101) that, in turn negatively affect the interpretation of this measure in a clinical setting. Furthermore, the MMSE used to assess cognitive status of our trainees showed rather high values for our sample, implying that there possibly was little room for improvement. Alternative explanations might relate to the training content. Regarding the 4 week time duration of the Exergame training component it would be reasonable to expect an observable change in cognition (35). However, the results might have been clearer when we would have adapted the training parameter frequency during the 4-week training course. Werner et al. (35) showed that rates of improvements slow down when no adaptations are implemented in for example the training frequency (35). More distinct effects may also be expected when well-considered exergames that content-wise are tailored to the training target group are used (102) as opposed to the training approach we used that applied an originally dancing arcade game geared toward leisure activities (103). Studies from our group showed that when purpose-developed games were used in older adult samples clear effects on executive functioning were observable (97, 98).

Falls-related self-efficacy was not significantly different between the two groups, a finding in line with former research (14). Fear of falling strongly relates to limitations of physical and social activities (104). It is assumed, however, that a significant change in falls-related self-efficacy requires more time than 8 weeks and may depend on other factors than those targeted in our intervention; e.g., psychological factors (105). Other intervention programmes for elderly that showed significant changes in falls-related self-efficacy (68, 106) had an intervention period duration of at least 12 weeks.

The intervention time of 8 weeks is consistent with recommendations (84) stating that acute effects of WBV can be expected after a 2-month intervention period. This recommendation is supported by other researchers (14, 86). Intervention periods of lesser duration did not lead to significant improvements (17, 43). The intervention period in the present study was enough to enhance physical function and cognitive performance.

## Limitations

This study has several limitations that should be acknowledged. The positive results seen in this study are exploratory in nature and should be a first indication for positive effects of technology-driven training. This because the sample size was small. The number needed per group for 80% power in a between-group comparison, using the SPPB as primary outcome, and with a focus on “substantial meaningful change” is 35 individuals per group (72). Therefore, this study should be replicated in a group of long-term care dwellers of enough size. However, the primary aim of pilot trials is not testing superiority (107). We can use the findings from our pilot trial to plan for a future main trial in which we plan the study design around a large effect. This would imply that for a future main trial we would need ≤44 individuals per arm for 90% power (107).

Another limitation is that, due to the many dropouts, the adherence rate was rather small (76.5%). Possible reasons for the many dropouts were the length of the intervention period. Some participants expressed to feel “being fixed” to the intervention plan and deemed three trainings per week was too much for them. One dropout referred to “inappropriate training time” as reason to stop. However, high attrition rates should be expected in long-term care (108), and should, consequently, be anticipated in the design of a future study. Due to influenza, other diseases or pain, four participants had to withdraw from the intervention. A third limitation was the intention to treat approach. As recommended in a systematic review on the quality of controlled trials (109), dropouts were analyzed in their original allocated group even if they did not participate in the entire intervention. This may have led to reduced variability of the data (68), but helped in avoiding selection and attrition bias (67, 109). With a fully powered future trial both intention-to-treat and per-protocol analysis should be considered. The fourth limitation relates to the cognitive effects of training. Based on the context of existing research we attributed the cognitive effects to exergaming. However, more recently there are some indications that WBV exercises might also affect cognition (110, 111).

## Conclusions

It can be concluded that an 8-week technology-driven intervention consisting of a combination of SR-WBV and EXDT, conducted three times a week, shows beneficial effects on physical functional and cognitive performance in long-term care. The results of this study provide evidence that training programmes for older adults who cannot participate in more conventional types of preventive training programmes, may consider using a technology-driven training approach as suitable alternative. Further research that aims to replicate the findings and that uses a sufficiently large sample size is warranted.

## Data Availability Statement

All datasets generated for this study are included in the article/Supplementary Material.

## Ethics Statement

The studies involving human participants were reviewed and approved by Ethics Committees from Cantons Bern and Zurich (EK ZH No 2014-0469). The patients/participants provided their written informed consent to participate in this study.

## Author's Note

Some of the material in this article first appeared in a thesis (112) from YB as fulfillment of the requirement for the Master of Science (M.Sc) in Physiotherapy education at Bern University of Applied Sciences. The thesis represents the only medium this information has appeared in, is in line with the author's university policy, and can be accessed online at: https://www.bfh.ch/gesundheit/de/ueber-das-departement-gesundheit.

## Author Contributions

SR and YB conceived the idea of this study. YB, EdB, HB, and SR participated in the conception and design of the study. EL managed the randomization. YB supervised the WBV and EXDT training sessions and SR the data collection. EL conducted the statistical analyses. YB and SR wrote the manuscript. YB, EdB, HB, EL, TH, and SR were involved in drafting or critically revising the manuscript. All authors read and approved the final manuscript.

## Conflict of Interest

The authors declare that the research was conducted in the absence of any commercial or financial relationships that could be construed as a potential conflict of interest.
