# Supplementary material for: Combining Stochastic Resonance Vibration With Exergaming for Motor-Cognitive Training in Long-Term Care; A Sham-Control Randomized Controlled Pilot Trial
Source: Front Med (Lausanne). 2020 Nov 30;7:507155. doi: 10.3389/fmed.2020.507155 (PMC7734185; doi:10.3389/fmed.2020.507155)
Supplement: Supplementary file 1 [file Data_Sheet_1.PDF]

Decision tree of the SR-WBV training program

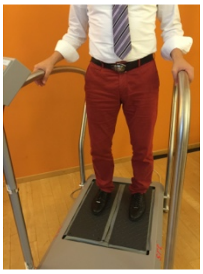

A: Normal stand & hold on both sides

if possible to stand in this position, then →

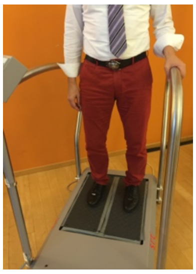

B: Normal stand & hold on one side

if possible to stand in this position over 20 seconds, then change to C. →

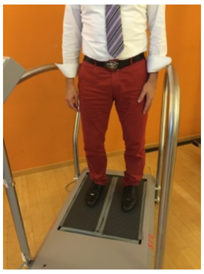

C: Normal stand without holding

if possible to stand in this position over 20 seconds, then change to D →

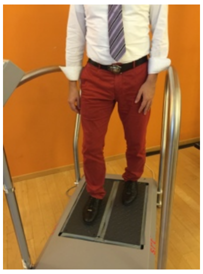

D: step position

if possible to stand in this position over 20 seconds, then change [illegible] →

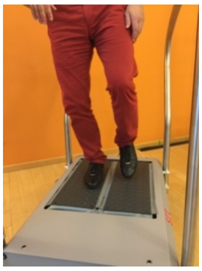

E: one leg stance

→

If possible to stand on one leg, then the frequency was increased about 1 Hz and starts with position A.
